# Supplementary material for: Memristor-based biomimetic compound eye for real-time collision detection
Source: Nat Commun. 2021 Oct 13;12:5979. doi: 10.1038/s41467-021-26314-8 (PMC8514515; doi:10.1038/s41467-021-26314-8)
Supplement: Supplementary file 1 — Supplementary Information [file 41467_2021_26314_MOESM1_ESM.pdf]

# Supplementary Informarion

**Memristor-based biomimetic compound eye for real-time collision detection**

Yan Wang[1,2], Yue Gong[1], Shenming Huang[1], Xuechao Xing[1], Ziyu Lv[1], Junjie Wang[1], Jiaqin Yang[1], Guohua Zhang[1], Ye Zhou[3] & Su-Ting Han[1,*]

*[1]Institute of Microscale optoelectronics and College of Optoelectronic Engineering, Shenzhen University, Shenzhen 518060, P. R. China*

*[2] Hefei Innovation Research Institute, School of Microelectronics, Beihang University, Hefei 230013, P. R. China*

*[3]Institute for Advanced Study, Shenzhen University, Shenzhen 518060, P. R. China*

*These authors contributed equally: Yan Wang, Yue Gong.*

**Correspondence and requests for materials should be addressed to S.-T.H. (e-mail: sutinghan@szu.edu.cn)*

**Supplementary Figures 1~33**
**Supplementary Notes 1~ 6**

## Supplementary Figures

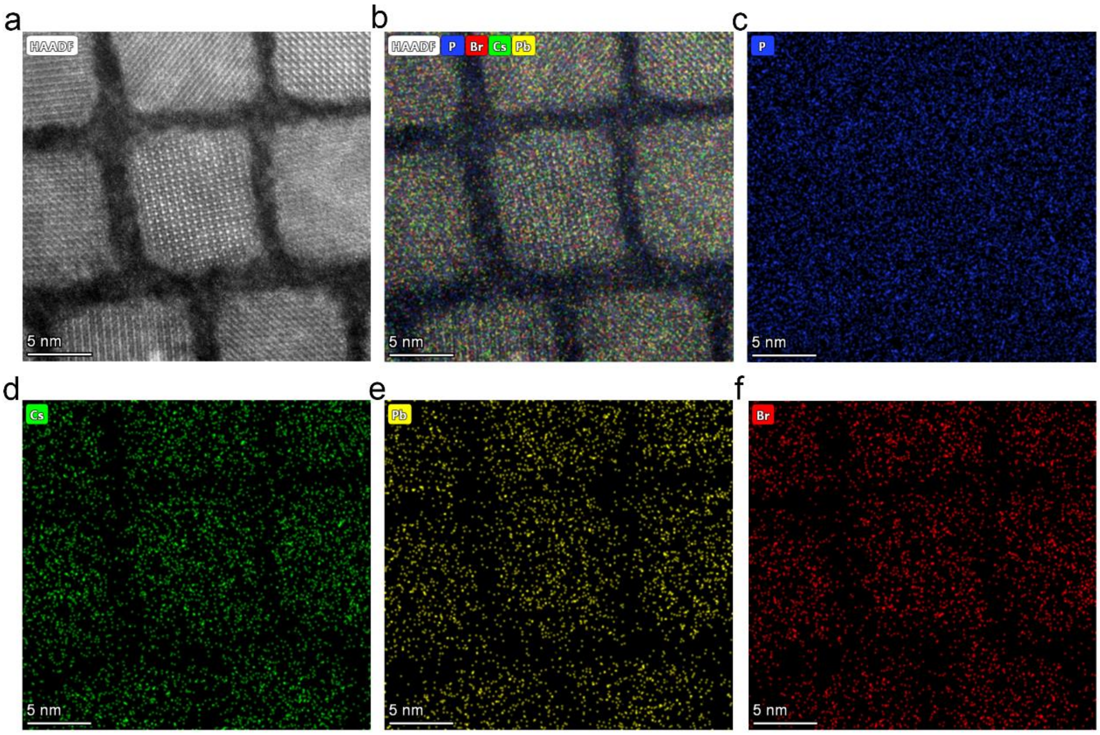


**Supplementary Figure 1. TEM characteristics of as-prepared FLBP-$CsPbBr_3$ samples. a** TEM image of FLBP-$CsPbBr_3$. Scale bar, 5 nm. **b-f** Energy-dispersive X-ray (EDX) elemental mapping profiles of selected areas of the FLBP-$CsPbBr_3$ nanocomposite with different element of (b) summary P, Cs, Pb, and Br, (c) P, (d) Cs, (e) Pb, and (f) Br.

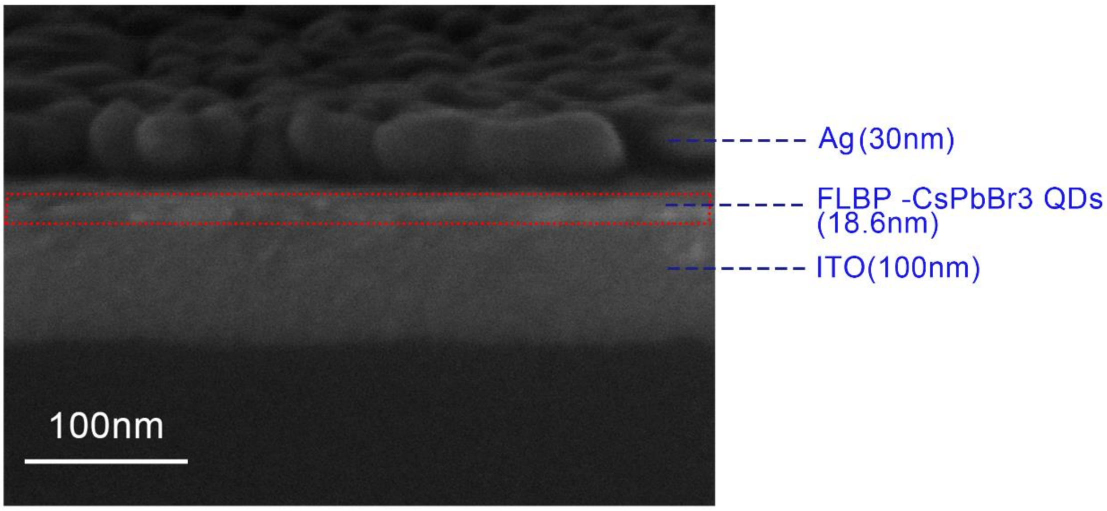


**Supplementary Figure 2. Cross-sectional SEM image of the vertical stack of the FLBP-$CsPbBr_3$ TSM.** Scale bar, 100 nm.

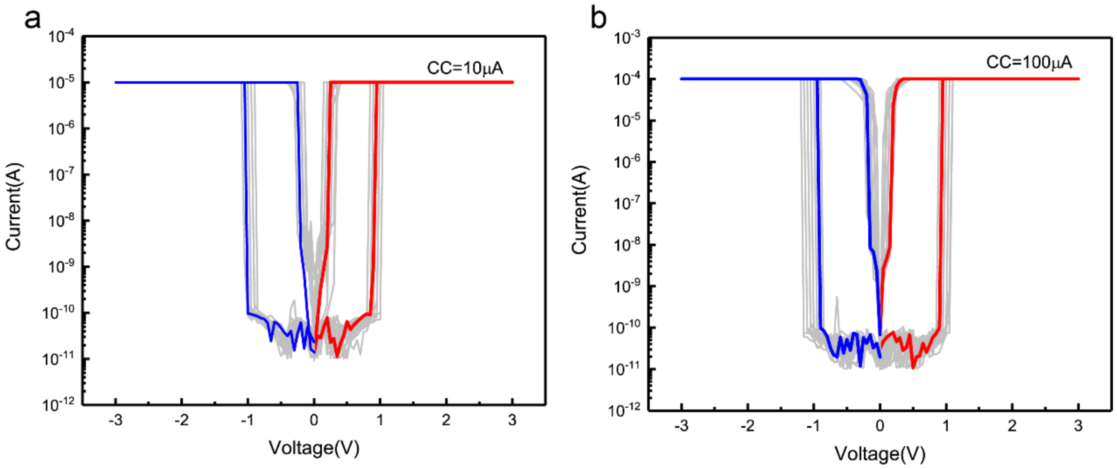


**Supplementary Figure 3.** Typical *I*-*V* characteristics of memristor under different current compliance (*I*cc) of (**a**) 10 μA, and (**b**) 100 μA.

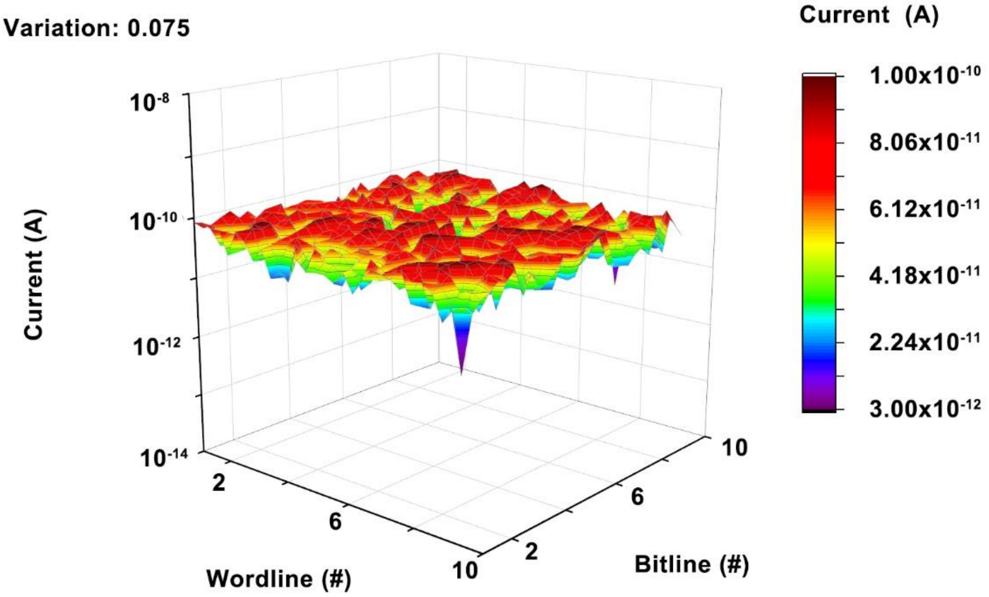


**Supplementary Figure 4.** Spatial HRS current uniformity of the 100 stable FLBP-$CsPbBr_3$ TSM devices.

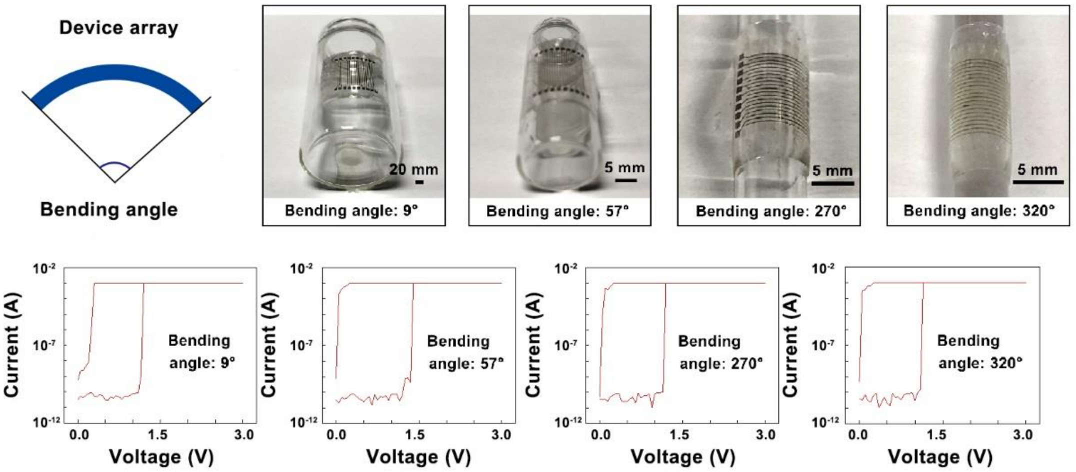


**Supplementary Figure 5.** Typical *I*-*V* curves with respect to the different curvatures of the TSM. The optical images of memristor with different bending angles are shown in the upper panel.

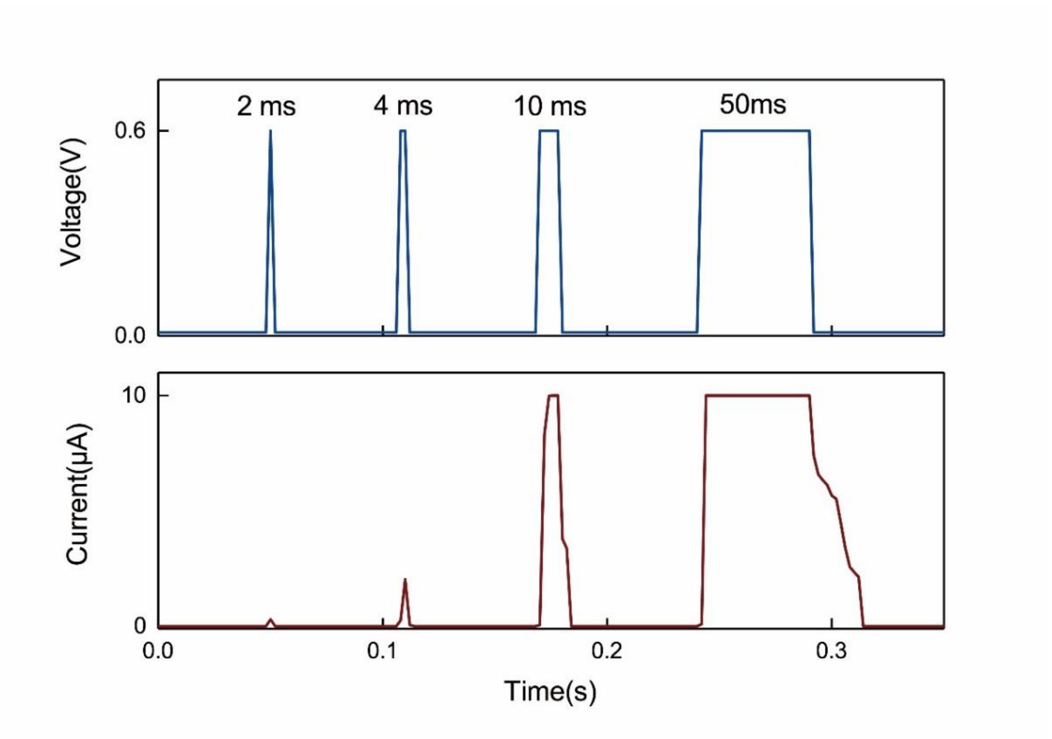


**Supplementary Figure 6.** Current response to a train of 0.6 V applied voltage pulses of variable durations (from 2 to 50 ms) (top panel) and response currents (bottom panel). A longer input pulse triggers a higher response current.

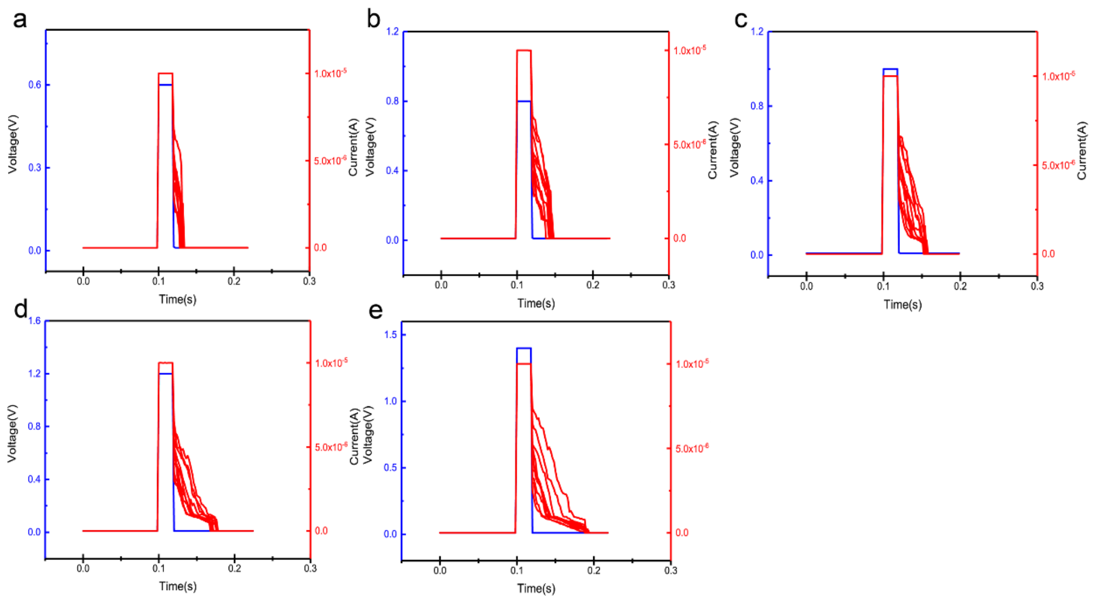


**Supplementary Figure 7. The transient resistive switching under electric pulses, exhibiting the varied relaxation time as a function of pulse voltage magnitude. a** 0.6 V, **b** 0.8 V, **c** 1.0 V, **d** 1.2 V, **e** 1.4 V. Here, the pulse width was fixed at 20 ms and a read voltage of 0.1 V was employed to monitor the current.

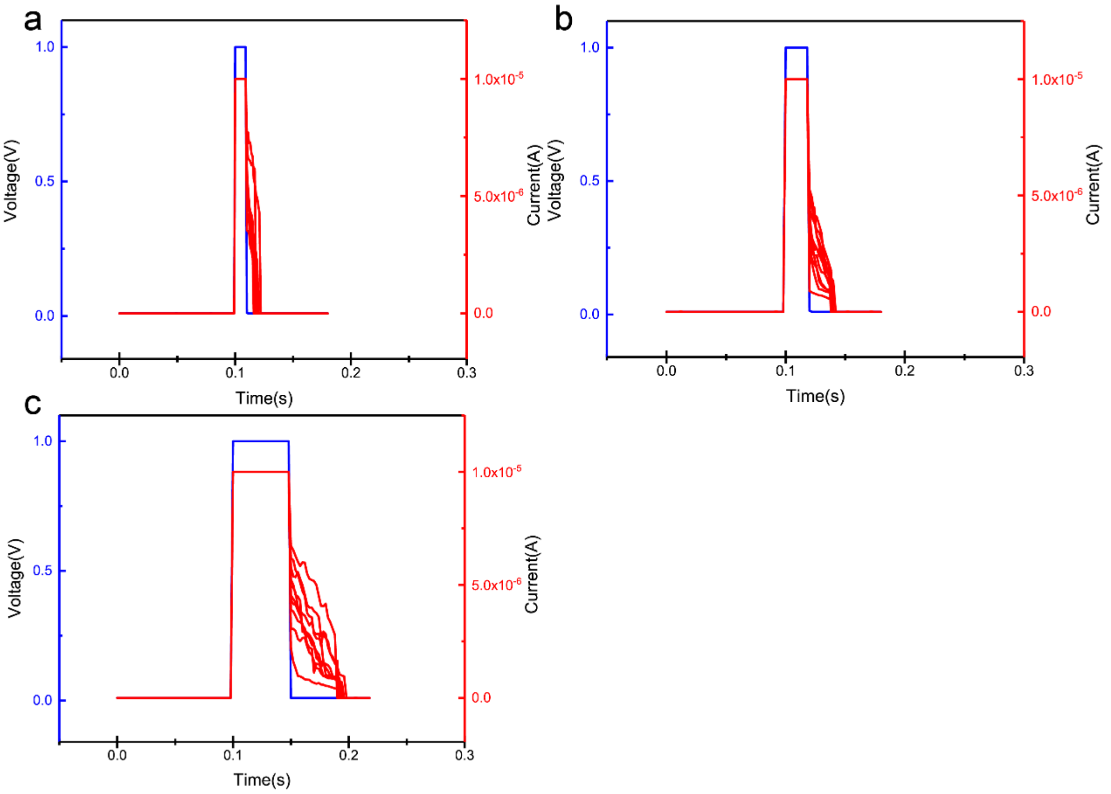


**Supplementary Figure 8. The transient resistive switching under electric pulses, exhibiting the varied relaxation time as a function of pulse width. a** 0.01 s, **b** 0.02 s, **c** 0.05 s. The pulse voltage was fixed at 1 V and a read voltage of 0.1 V was employed to monitor the current.

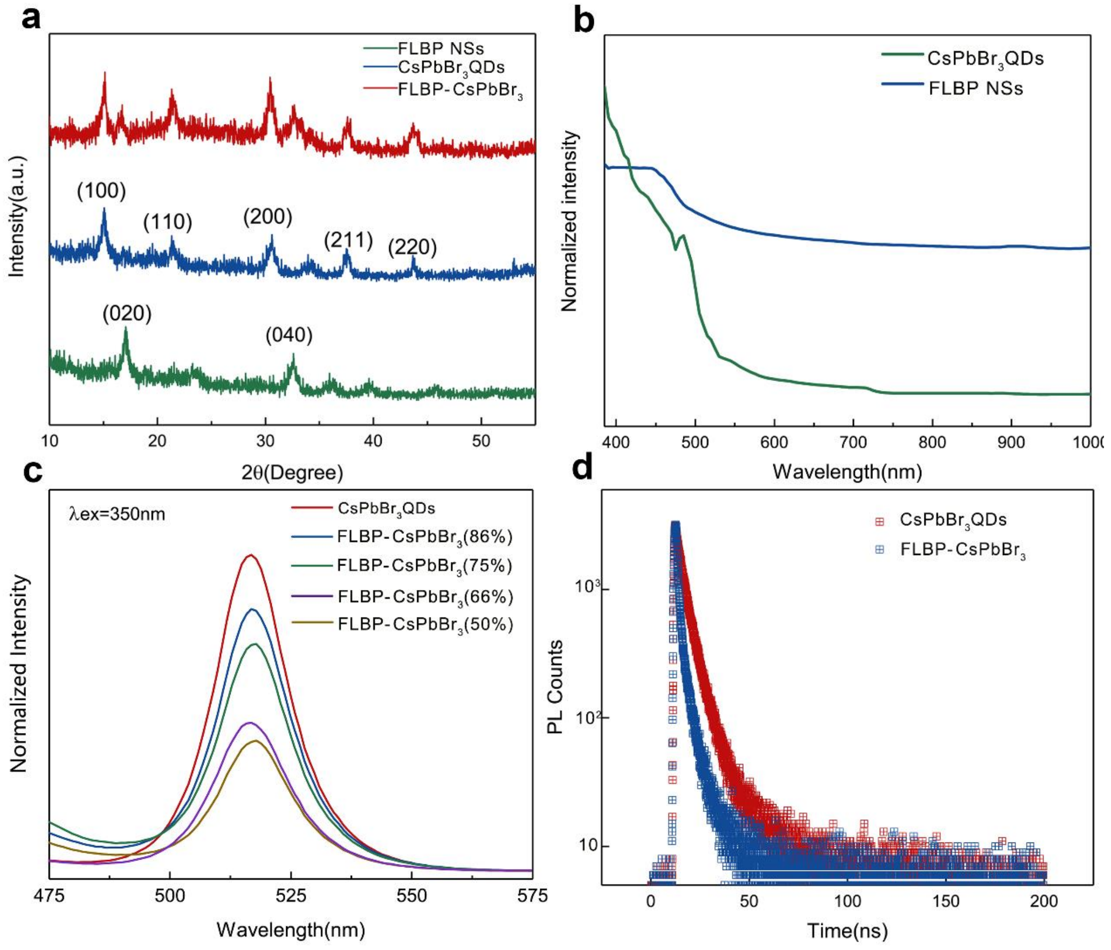


**Supplementary Figure 9. a** X-ray diffraction (XRD) patterns of FLBP NSs, $CsPbBr_3$ QDs and FLBP-$CsPbBr_3$. **b** UV-vis measurements of the FLBP NSs-based film and $CsPbBr_3$ QDs-based film. **c** Photoluminescene (PL) spectra of FLBP-$CsPbBr_3$ with variable concentrations of $CsPbBr_3$ QDs. **d** Time resolved PL (TRPL) spectra of FLBP-$CsPbBr_3$.

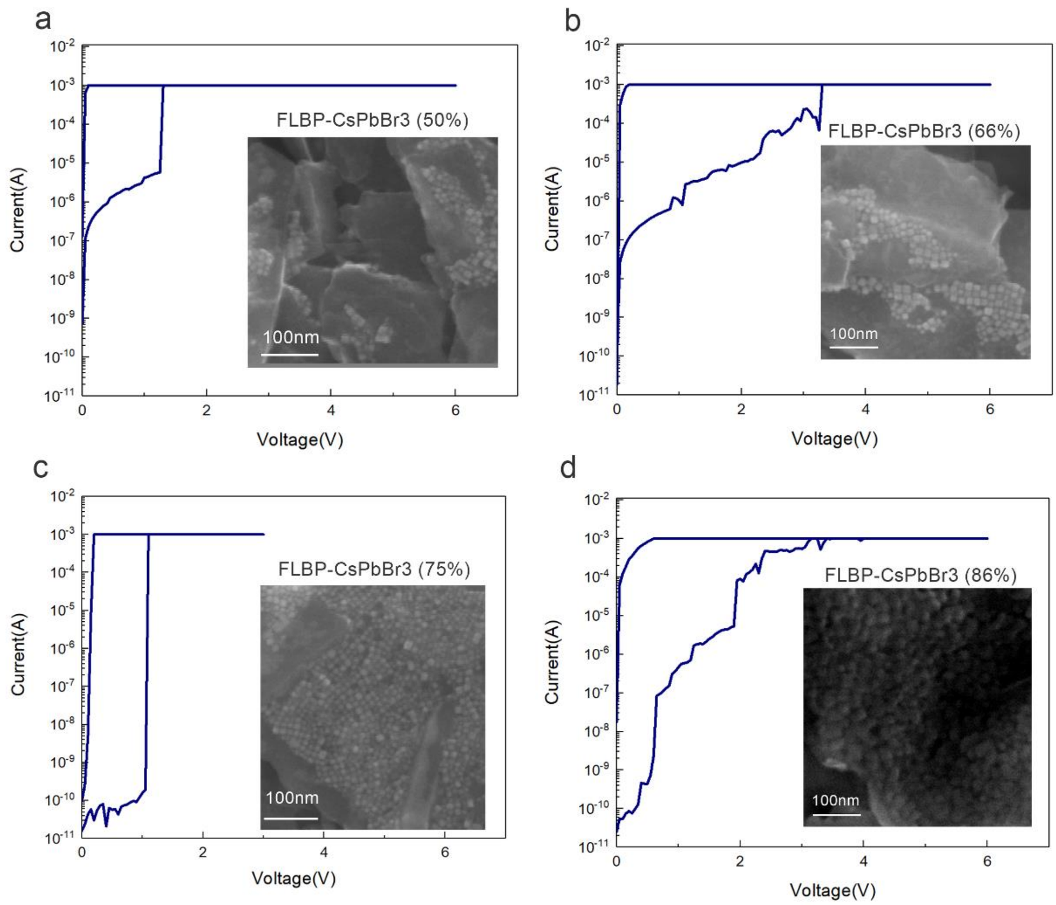


**Supplementary Figure 10. The *I-V* characteristic curves of different FLBP-$CsPbBr_3$ nanocomposite. a** FLBP-$CsPbBr_3$ (50%). **b** FLBP-$CsPbBr_3$ (66%). **c** FLBP-$CsPbBr_3$ (75%). **d** FLBP-$CsPbBr_3$ (86%). The relative higher resistive switching performance of FLBP-$CsPbBr_3$ (75%) based TSM suggests a better nanocomponent for the subsequent study. For the simplicity of description, FLBP-$CsPbBr_3$ (75%) is thus abbreviated as FLBP-$CsPbBr_3$ in the manuscript.

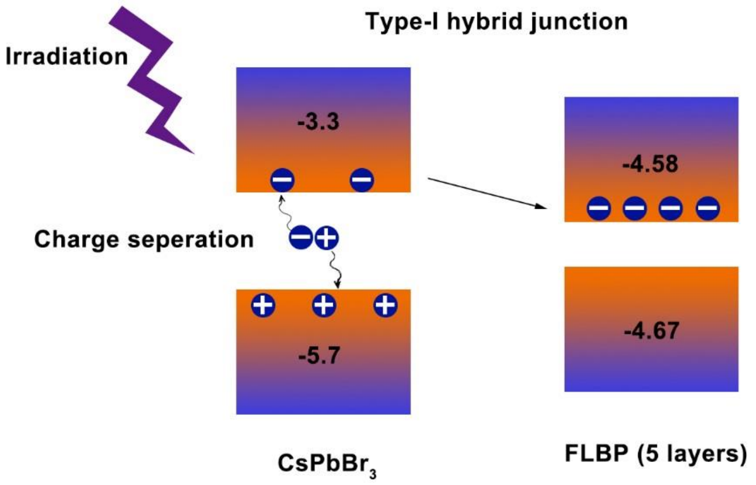


**Supplementary Figure 11.** Sketch of the conduction and valence band profiles and the electron-hole dynamics under irradiation.

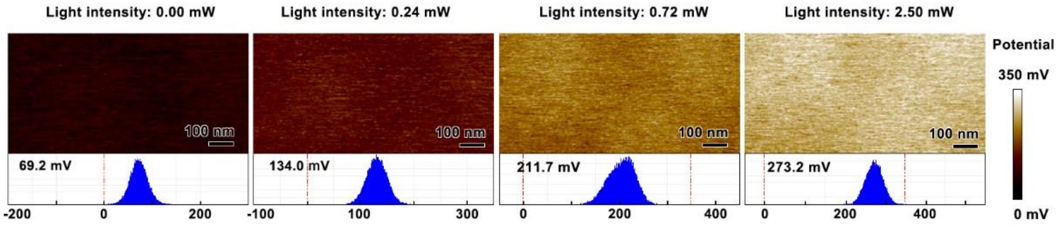


**Supplementary Figure 12.** Surface potential of the FLBP-$CsPbBr_3$ layer under different light illumination (fixed light wavelength: 365 nm) recorded by in situ AFM electrical nano-technology (scale bar, 100 nm). The lower panel shows the respective surface potential profile.

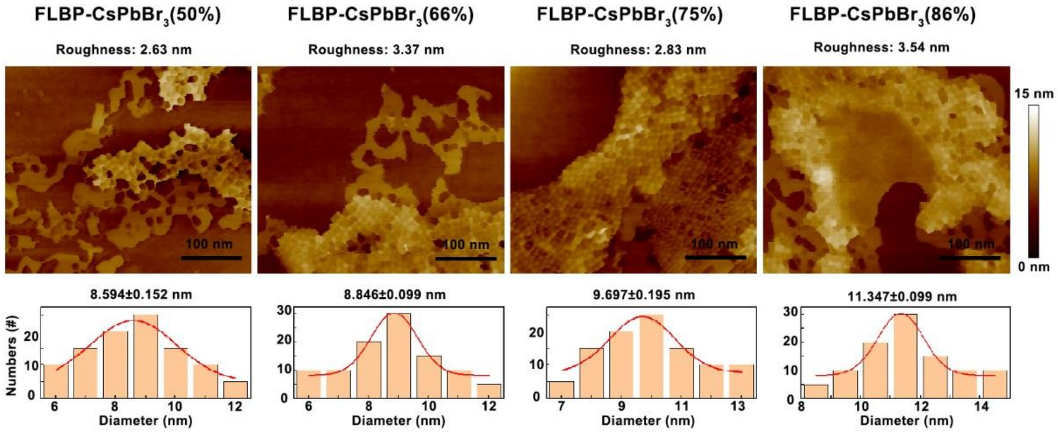


**Supplementary Figure 13.** Demonstration of the roughness of the different FLBP-$CsPbBr_3$ film and the grain size of assembled $CsPbBr_3$ QDs.

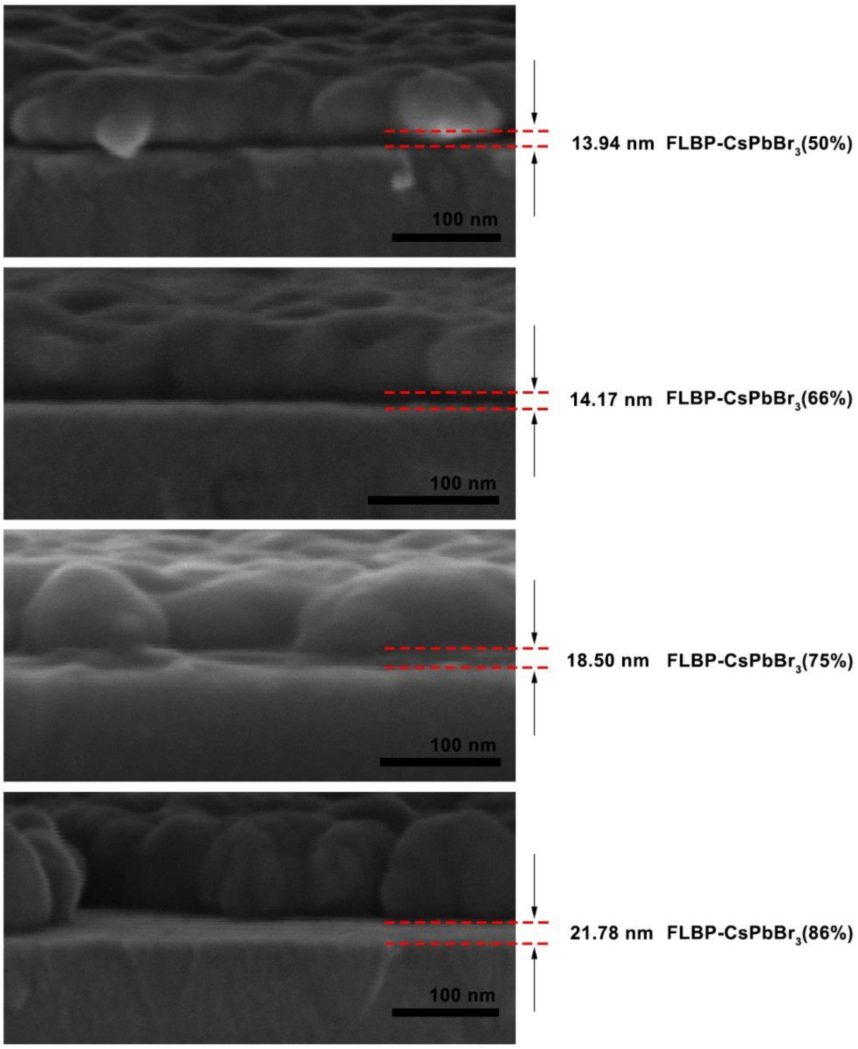


**Supplementary Figure 14.** Cross-sectional SEM image of the vertical stack of the TSM fabricated with FLBP-$CsPbBr_3$(50%), FLBP-$CsPbBr_3$(66%), FLBP-$CsPbBr_3$(75%) and FLBP-$CsPbBr_3$(86%).

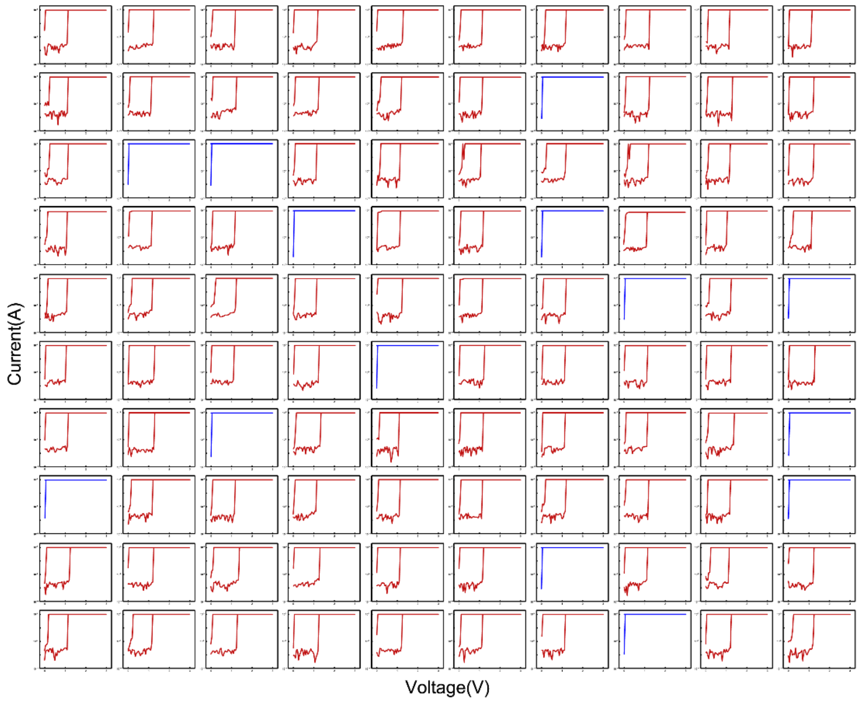


**Supplementary Figure 15.** *I*-*V* characteristics of the first 100 FLBP-$CsPbBr_3$ TSM devices in a 20×20 crossbar array. All the *I*-*V* curves were obtained by sweeping the voltage in the sequence of 0 V → 3 V → 0 V.

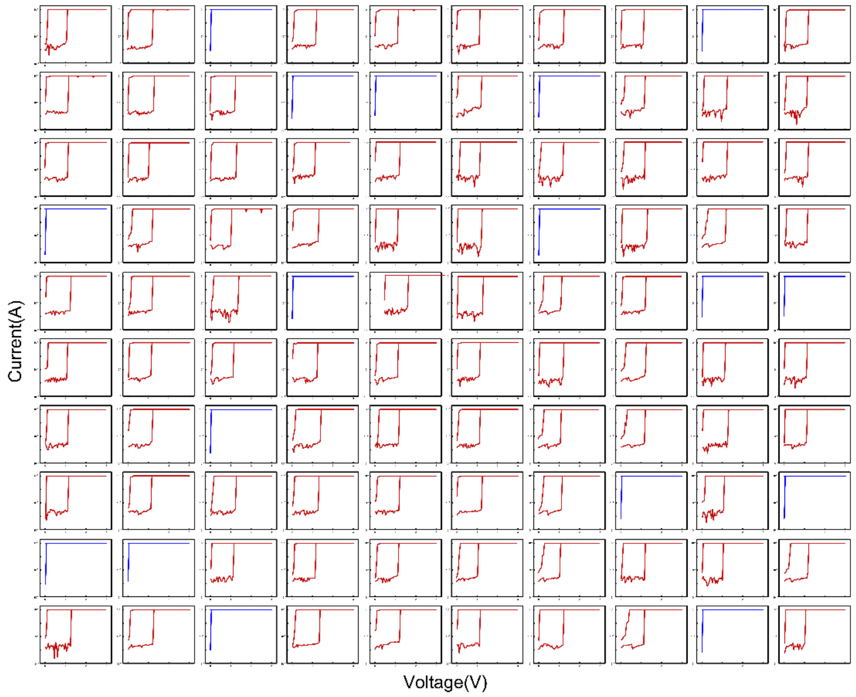


**Supplementary Figure 16.** *I*-*V* characteristics of the second 100 FLBP-$CsPbBr_3$ TSM devices in a 20 × 20 crossbar array. All the *I*-*V* curves were obtained by sweeping the voltage in the sequence of 0 V → 3 V → 0 V.

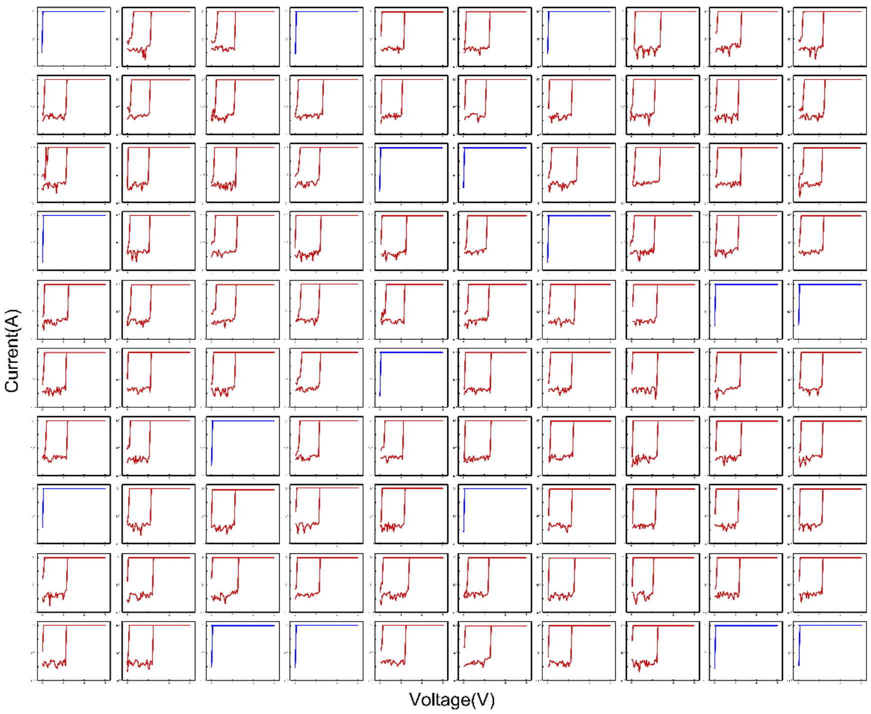


**Supplementary Figure 17.** *I*-*V* characteristics of the third 100 FLBP-$CsPbBr_3$ TSM devices in a 20 × 20 crossbar array. All the *I*-*V* curves were obtained by sweeping the voltage in the sequence of 0 V → 3 V → 0 V.

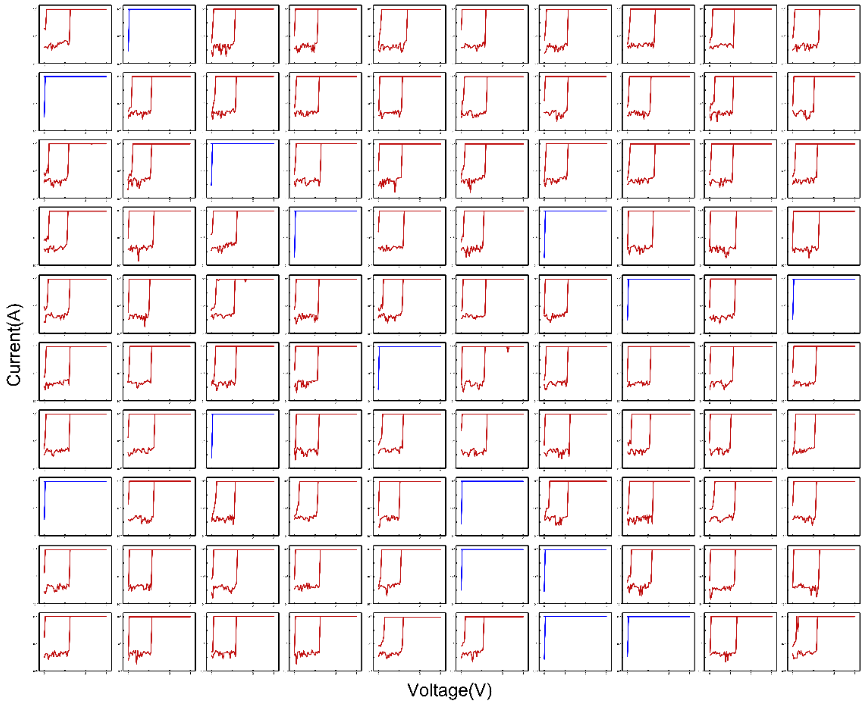


**Supplementary Figure 18.** *I*-*V* characteristics of the fourth 100 FLBP-$CsPbBr_3$ TSM devices in a 20 × 20 crossbar array. All the *I*-*V* curves were obtained by sweeping the voltage in the sequence of 0 V → 3 V → 0 V.

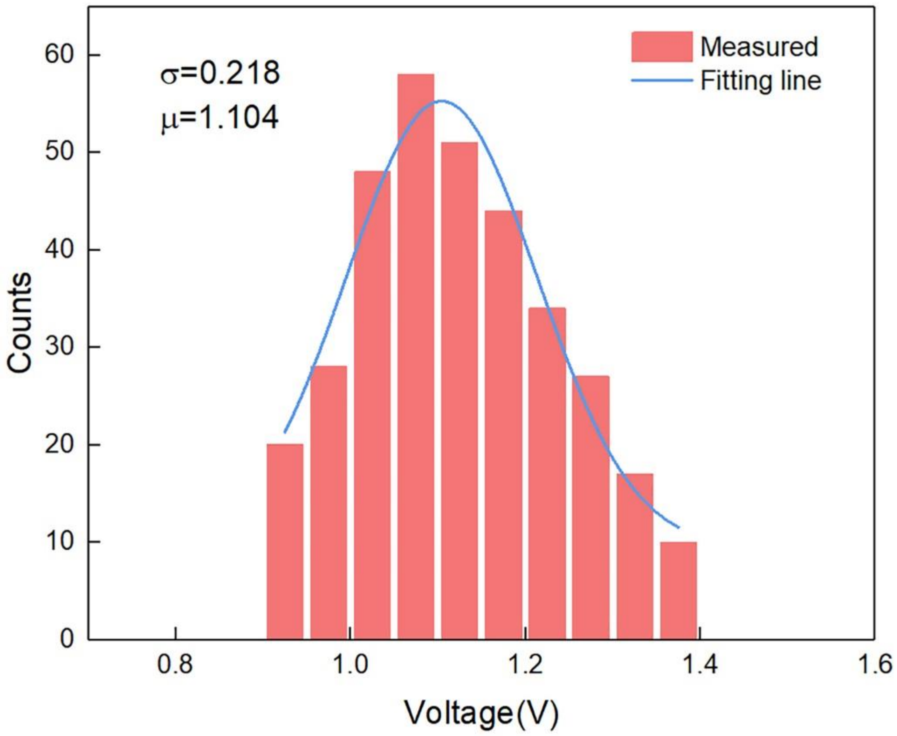


**Supplementary Figure 19.** Histogram of $V_{th}$ of FLBP-$CsPbBr_3$ TSM devices in a 20 × 20 crossbar array under positive sweep with Gaussian fitting.

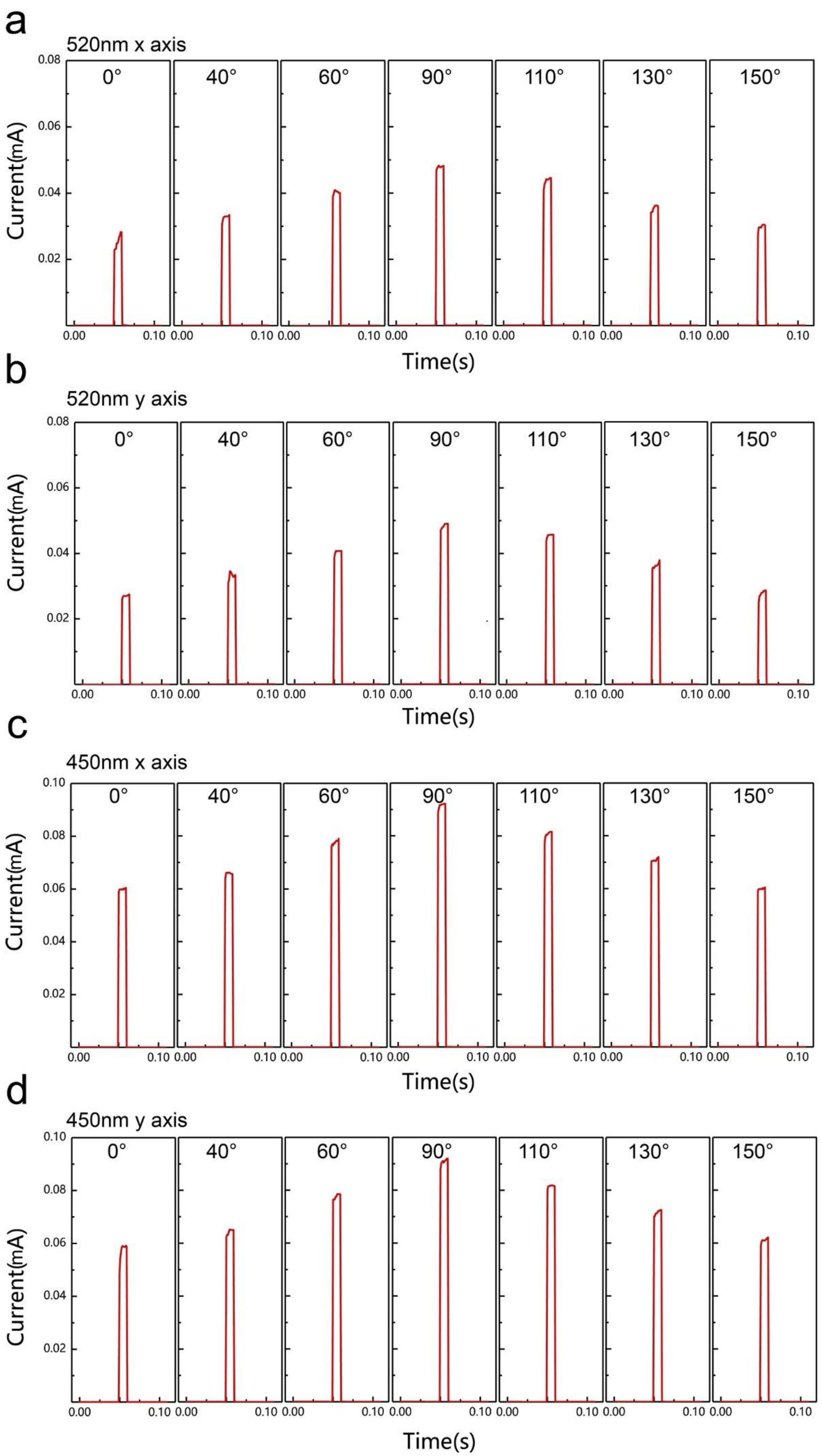
a
520nm x axis
0° 40° 60° 90° 110° 130° 150°
Current(mA)
Time(s)
b
520nm y axis
0° 40° 60° 90° 110° 130° 150°
Current(mA)
Time(s)
c
450nm x axis
0° 40° 60° 90° 110° 130° 150°
Current(mA)
Time(s)
d
450nm y axis
0° 40° 60° 90° 110° 130° 150°
Current(mA)
Time(s)

**Supplementary Figure 20. Angle-sensitive responses in the TSM for optic pulsed tests. a** Current response along *x* direction (520 nm wavelength, 0.72 mW power, 10 ms pulse width). **b** Current response along *y* direction (520 nm wavelength, 0.72 mW power, 10 ms pulse width). **c** Current response along *x* direction (450 nm wavelength, 0.72 mW power, 10 ms pulse width). **d** Current response along *y* direction (450 nm wavelength, 0.72 mW power, 10 ms pulse width).

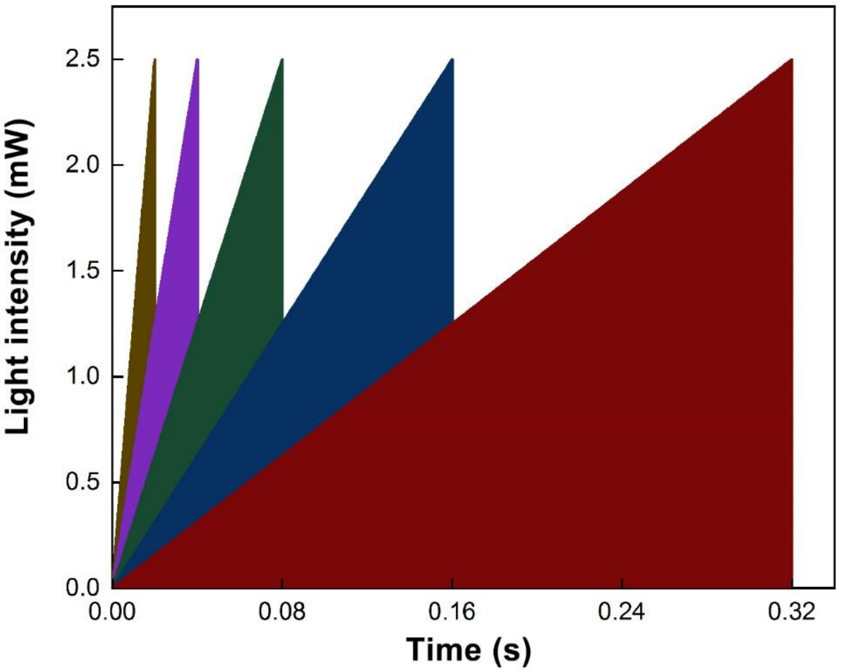


**Supplementary Figure 21.** Monotonic increase in the light power ramped from 0.00 to 2.50 mW for different time interval determing the approaching speed of the object.

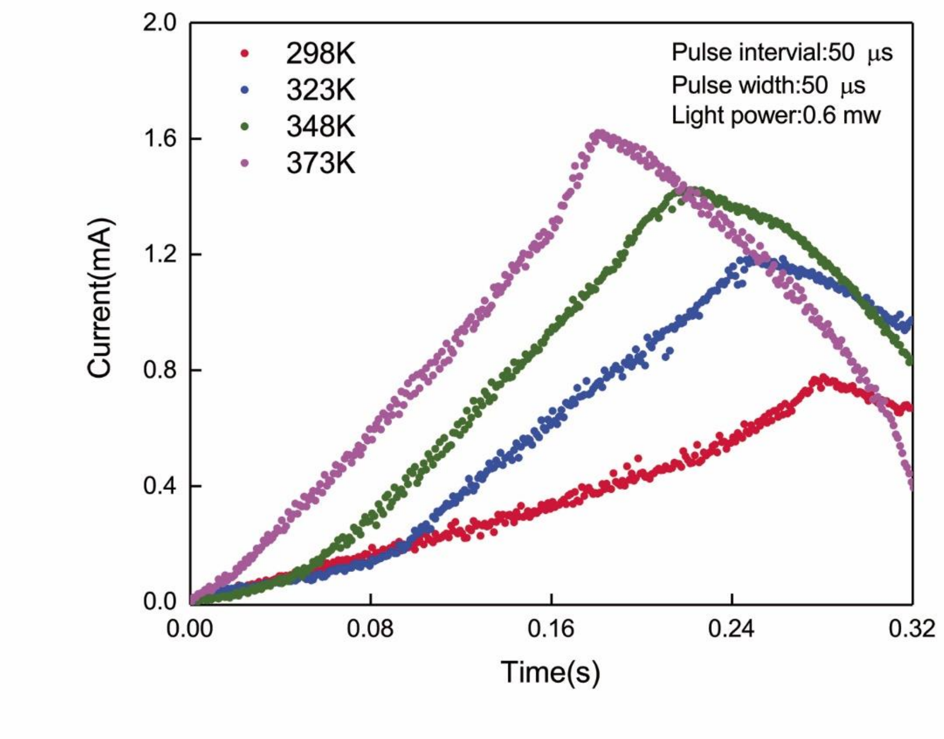


**Supplementary Figure 22.** Excitatory and inhibitory response of the device to a looming light stimulus with simultaneously applied programming electronic pulses under diverse temperatures ranging from 298 K to 373 K (0.2 V voltage pulse, 50 μs duration, 50 μs interval).

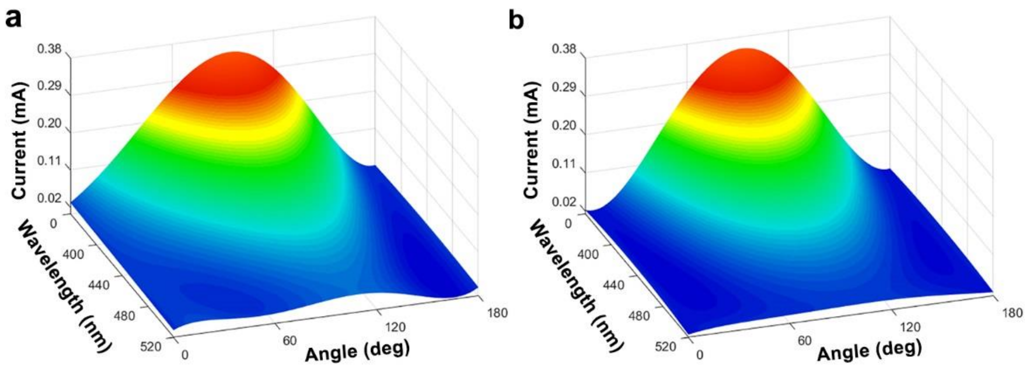


**Supplementary Figure 23.** The current as a function of light wavelength and the incident angle along *x* direction (a) and *y* direction (b).

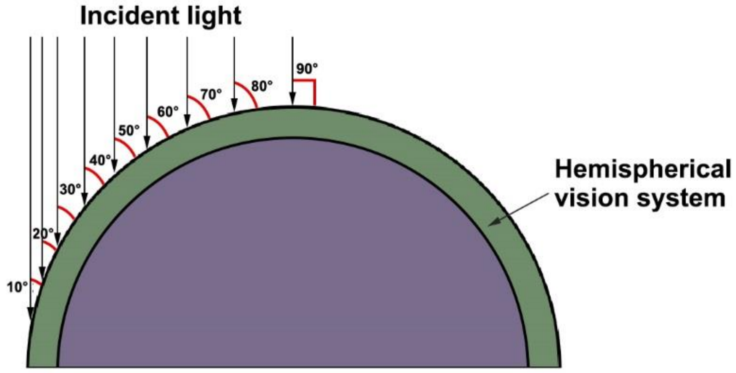


**Supplementary Figure 24.** Specific 9 positions with incident angles of 10°, 20°, 30°, 40°, 50°, 60°, 70°, 80° and 90° for the subsequent collision avoidance investigation.

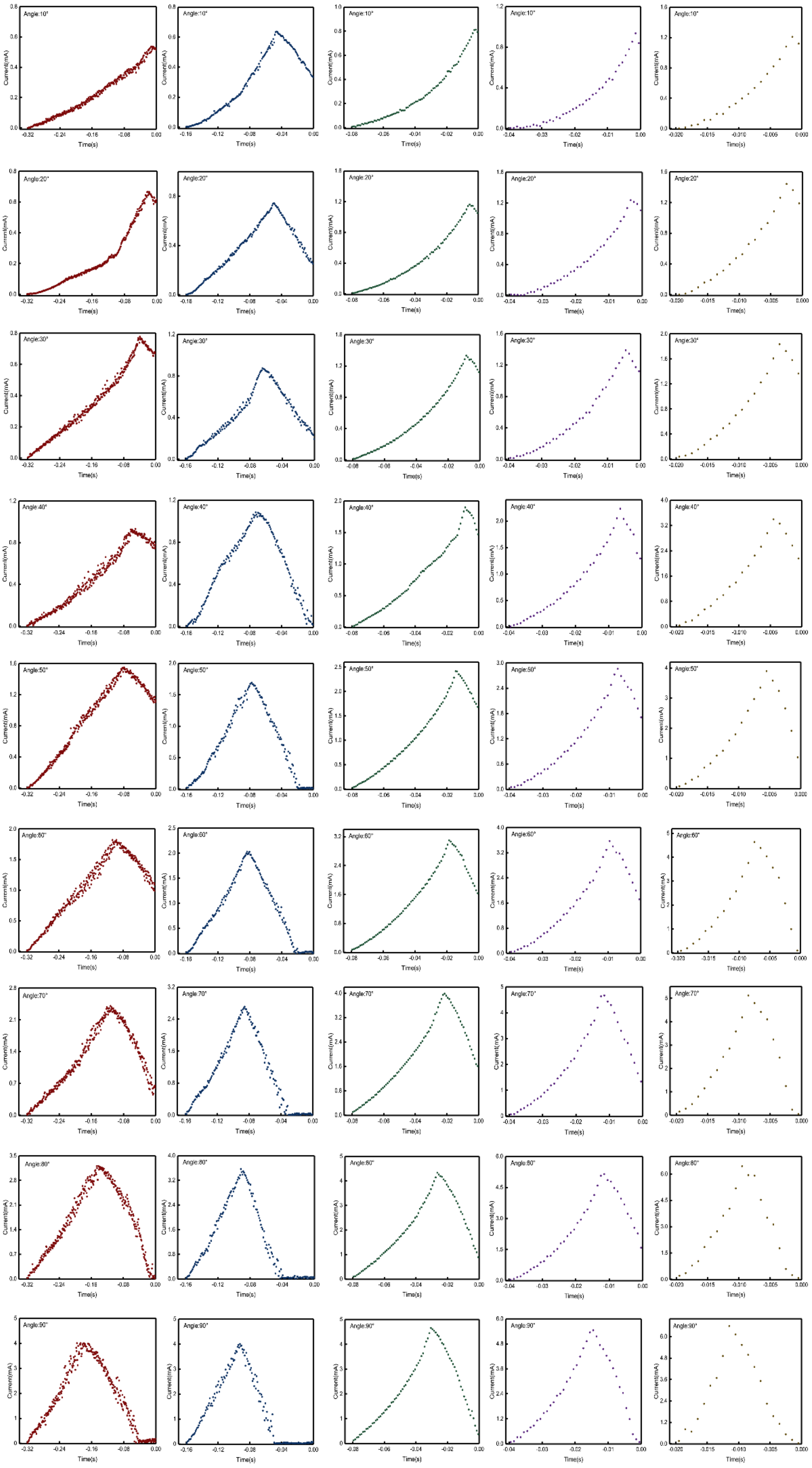


**Supplementary Figure 25.** Output current of the different positioned FLBP-$CsPbBr_3$TSM device in response to different looming object speed.

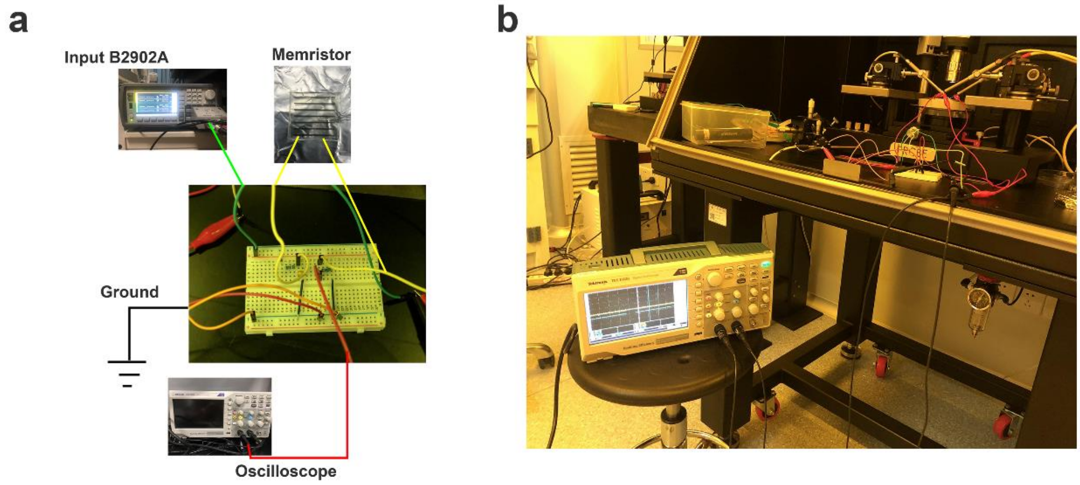


**Supplementary Figure 26. Experimental setup for FLBP-CsPbBr$_3$ TSM based IF model neuron. a** The neuron circuit test platform of the implemented neuron. Note that the internal voltage change of the circuit is monitored with an oscilloscope. **b** Photo of the test setup with the IF model neuron.

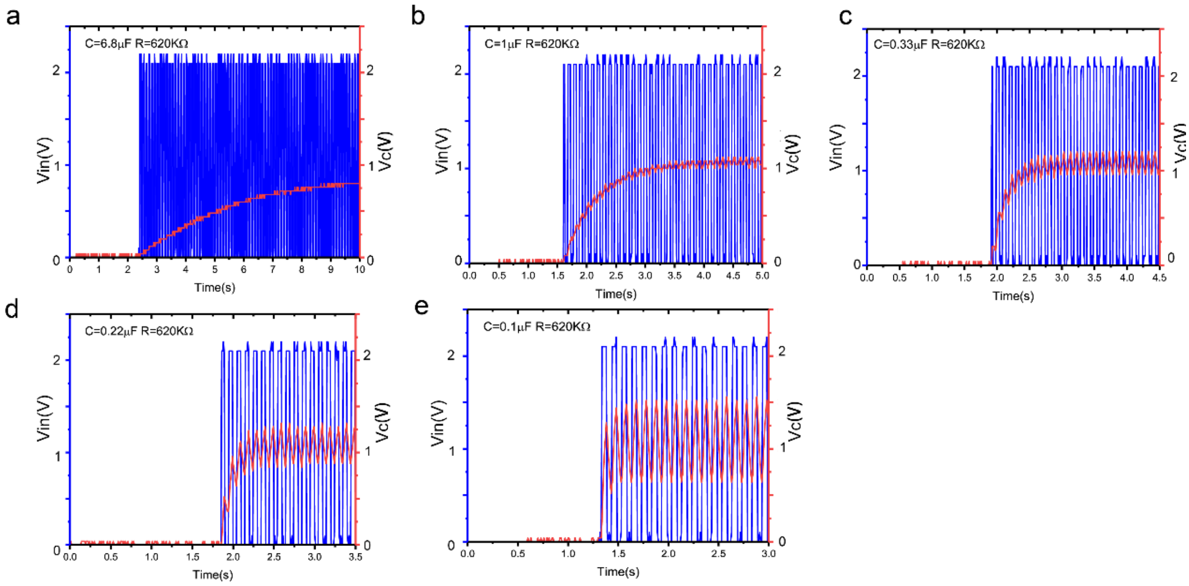


**Supplementary Figure 27. Charging response curves of IF model neuron based on different capacitors.** (Pulse voltage: 2 V, pulse width: 50 ms, $R_1$=620 KΩ) **a** $C_p$=6.8 μF. **b** $C_p$=1 μF. **c** $C_p$=0.33 μF. **d** $C_p$=0.22 μF. **e** $C_p$=0.1 μF.

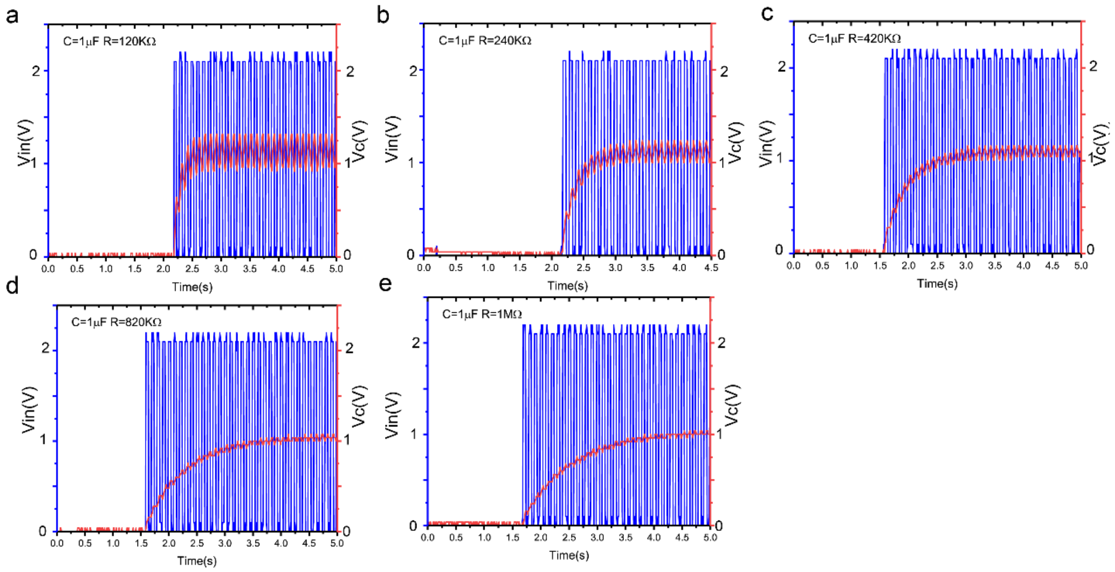


**Supplementary Figure 28. Charging response curves of IF model based on different resistors.** (Pulse voltage: 2 V, pulse width: 50 ms, $C_p$=1μF) **a** $R_1$=120 KΩ, **b** $R_1$=240 KΩ, **c** $R_1$=420 KΩ, **d** $R_1$=820 KΩ, **e** $R_1$=1 MΩ.

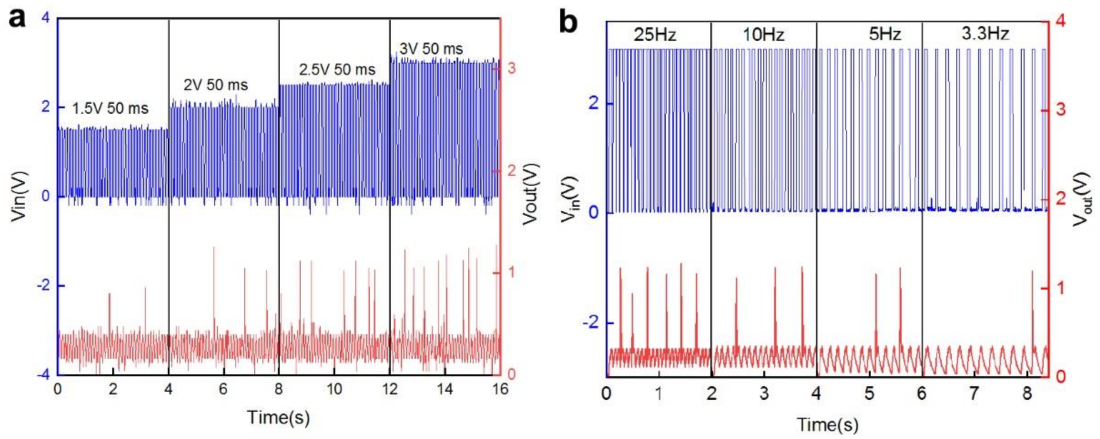


**Supplementary Figure 29. The voltage response of TSM neuron evoked by repetitive pulses. a** Experimental firing effect in TSM neuron as a function of the pulse magnitude. The firing probability increases from 0.05, 0.075, 0.15 to 0.225 as the pulse magnitude increases from 1.5, 2, 2.5 to 3 V, respectively. **b** Experimental firing effect in TSM neuron as a function of the pulse frequency. The firing probability increases from 0.083, 0.154, 0.158 to 0.222 as the pulse magnitude increases from 3.3, 5, 10 to 25 Hz, respectively.

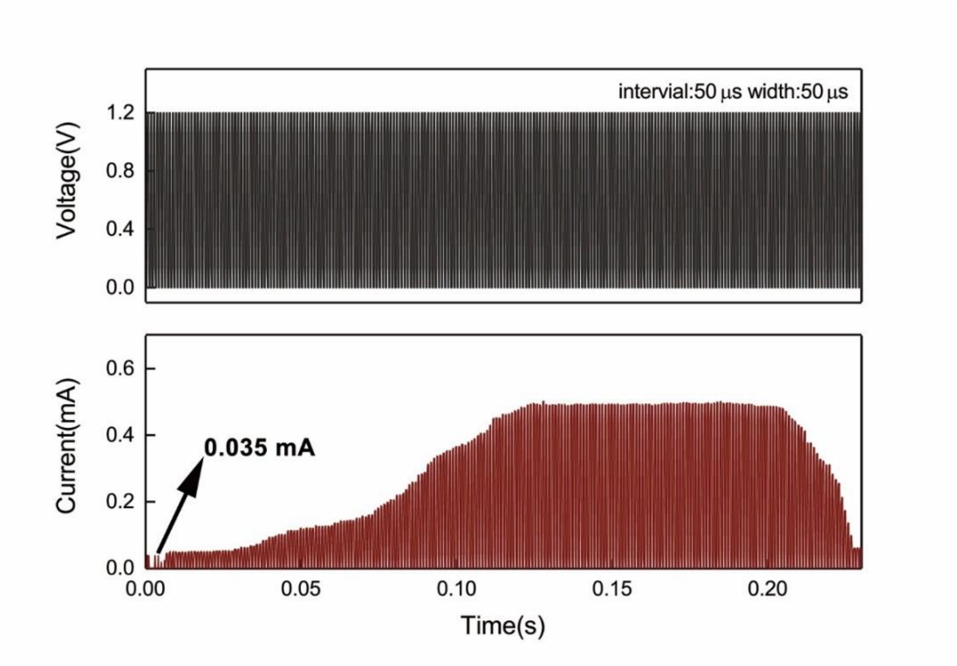


**Supplementary Figure 30.** Output currents measured in response to the programming pulse trains with an interval of 50 μs and width of 50 μs.

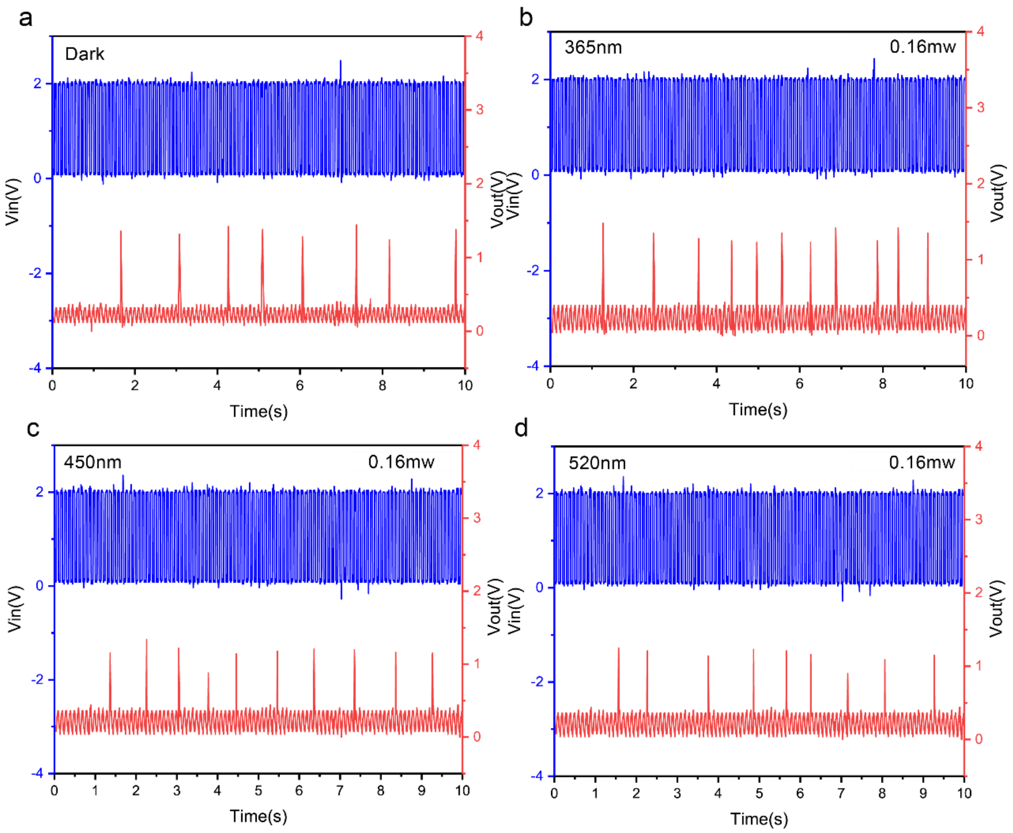


**Supplementary Figure 31. The voltage response of TSM neuron evoked by repetitive pulses (2 V, 50 ms) under different light irradiation (power: 0.16 mW). a** dark, **b** 365 nm, **c** 450 nm, **d** 520 nm.

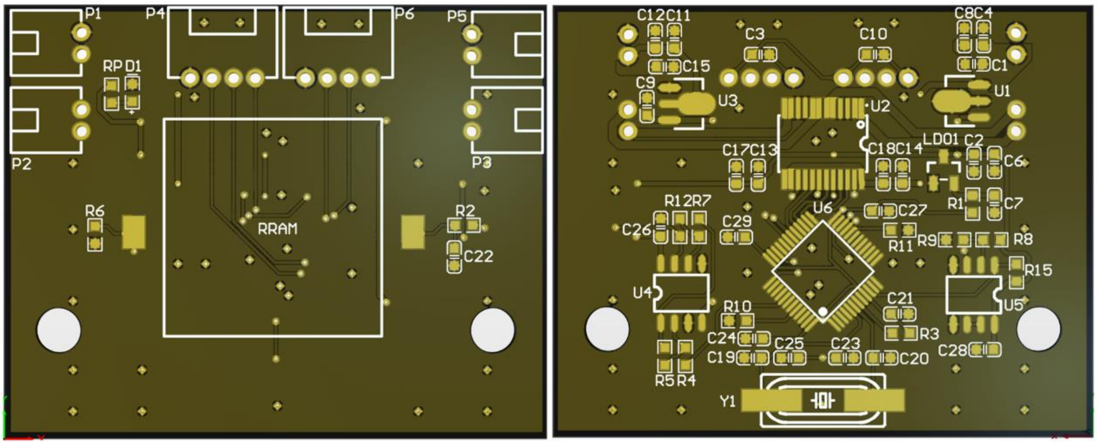


**Supplementary Figure 32.** Printed circuit board (PCB) circuit diagram of car robot to make decision in the motion trajectory with implemented FLBP-$CsPbBr_3$ TSM device.

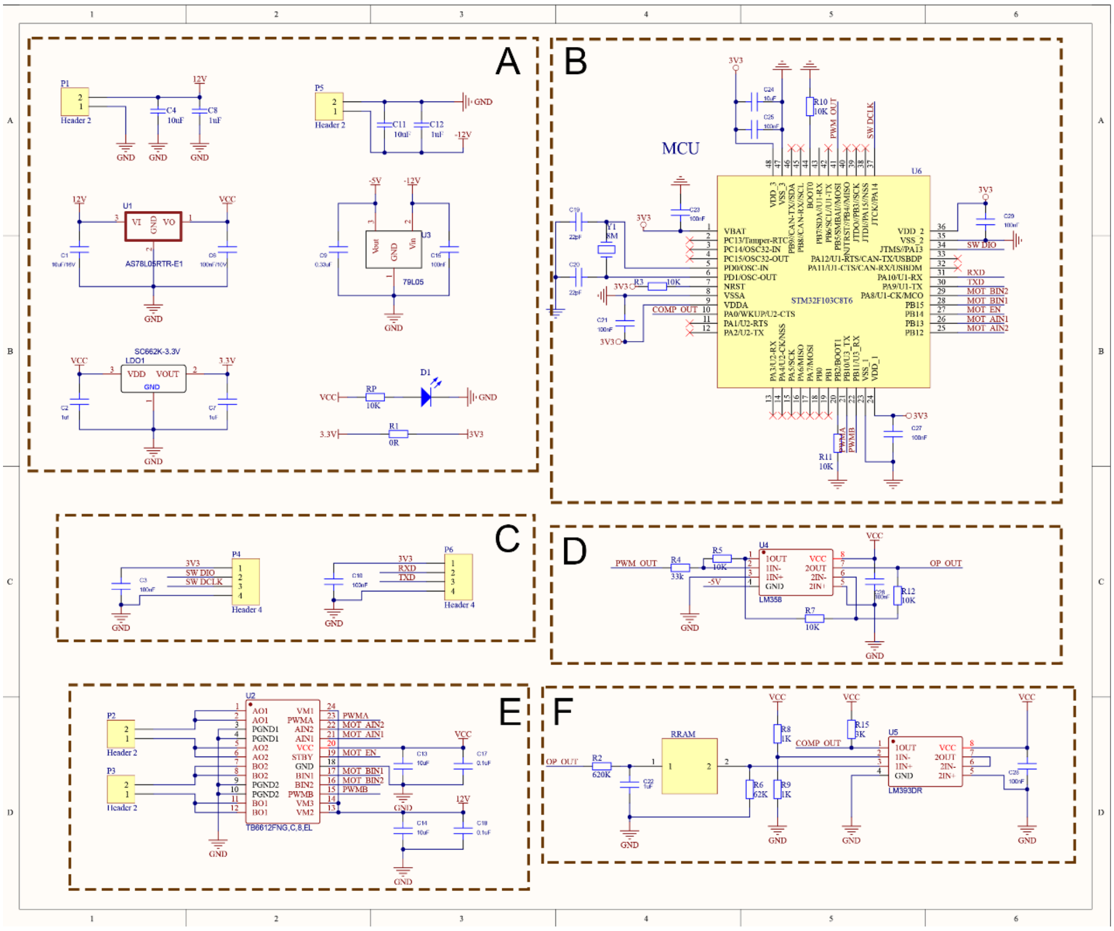


**Supplementary Figure 33. Schematics of circuit components. Schematics of circuit components. The schematic diagram is divided into 6 areas.**

The circuit design mainly includes the following parts:

Zone A named power supplement part: the 12 V input voltage is converted to 5 V by the 78L05 chip, the 5 V voltage is converted to 3.3 V by the SC662K chip. Among them D1 is the power indicator light.

Zone B named controller part: STM32 and its peripheral circuit control the corresponding

output pulse waveform and control the drive circuit according to the input voltage signal.

Zone C named serial port part: download the program and monitor the running status of the program

Zone D named amplifier part: convert the controller output voltage from 3.3 V to 1 V.

Zone E named driving circuit: receive the signal from the controller and controls the car to go straight or turn.

Zone F named light sensor circuit: The pulse signal output by the controller is passed through memristor to obtain the output voltage spiking frequency, and the output voltage spiking frequency is compared with the reference spiking frequency through a comparator. Finally, the output voltage spiking frequency is transmitted to the controller. If the output voltage spiking frequency of the comparator is high than 2.5 Hz, the controller controls the car to turn.

Working principle: The Zone B controller outputs a 3.3 V pulse waveform and transmits it to the Zone D amplifier, and the amplifier converts the pulse into 1V pulse and inputs it to the Zone F light sensor circuit. The light sensor circuit uses the output voltage obtained by the comparator to determine whether it is fire by comparing the relationship between the output voltage spiking frequency and 2.5 Hz, and counts the number of firing through the internal counter of the single-chip microcomputer to determine whether it reaches the avoidance frequency. Finally, the result is sent back to the control circuit, and the control circuit controls the driving circuit to go straight or turn according to the Zone E.

## Supplementary Note

### Supplementary Note 1

Based on the 100 stable TSM devices, we characterize the device-to-device variation by measuring the HRS current of 100 samples. As shown in Supplementary Fig. 4, the standard deviation of the HRS current was as low as 0.075. The narrow distribution of HRS current indicates high reproducibility of our device. While the corresponding *I-V* characterization with varied bending angles was provided in Supplementary Fig. 5. The stable threshold switching performance as the device array is bending from 9° to 320° demonstrates the feasibility of the flexible applications.

### Supplementary Note 2

XRD pattern of as-synthesized $CsPbBr_3$ QDs with a typical cubic structure (JCPDS No. 54–0752) and a typical peak at 30.68° corresponding to (200) reflection is shown in Supplementary Fig. 9a. All the indexed peaks of the FLBP-$CsPbBr_3$ can be well assigned with the $CsPbBr_3$ QDs and FLBP NSs, suggesting successful formation of the self-assembled nanocomposite. As BP is a promising candidate for optoelectronics applications owing to its exclusive anisotropic puckered configuration and existence of a direct band gap from 0.3 eV of bulk to 2 eV of monolayer. While quantum structured $CsPbBr_3$ perovskites is recently at the focus of enormous scientific attention in the sight of their astonishing optical characteristics. According to the UV-visible spectra (Supplementary Fig. 9b) and static/dynamic photoluminescence spectra (Supplementary Fig. 9c,d), 0D-2D FLBP-$CsPbBr_3$ nanocomposite exhibit an absorption band with a broad maximum between 450 and 490 nm and quenched luminescence compared with pristine $CsPbBr_3$ QDs and FLBP NSs. The results of optical spectroscopy indicate that the light induces electron transport from the photo-excited $CsPbBr_3$ QDs to FLBP NSs. Then the injected electrons will be trapped in FLBP owing to its large surface area and high carrier mobility, leading to the accumulation of electrons in FLBP and formation of internal electrical field. It is worth noting that the conductivity of the FLBP-$CsPbBr_3$ nanocomposite depends on the component of $CsPbBr_3$ nanocomposites, which is compared in Supplementary Fig. 10.

### Supplementary Note 3

As shown in the Supplementary Fig. 11, the conduction band (valence band) of the few layered FLBP NSs and $CsPbBr_3$ QDs is -4.58 eV (-4.67 eV) and -3.3 eV (-5.7 eV), respectively, hence the hybrid junction is expected to be type-I with a band offset of 1.28 eV (The electronic band structure of the FLBP varies with the layer numbers. Here we take 5 layered FLBP as an example). The large band offset induces the separation of the photo-generated electron-hole pairs in $CsPbBr_3$ and transportation of the electrons to FLBP. Additionally, the photo-induced electron transfer from $CsPbBr_3$ to FLBP was further confirmed by the KPFM measurements in Supplementary Fig. 12.

### Supplementary Note 4

The average roughness of FLBP-$CsPbBr_3$ layer varies with the concentration of $CsPbBr_3$. As shown in Supplementary Fig. 13, the FLBP-$CsPbBr_3$(50%) and FLBP-$CsPbBr_3$(75%) exhibit roughness of 2.63 nm and 2.83 nm, respectively, suggesting the better quality of the FLBP-$CsPbBr_3$ layer. Relative higher proportion of FLBP in the FLBP-$CsPbBr_3$ (50%) exhibit higher film conductivity. In addition, the film thicknesses of the samples with different concentration of $CsPbBr_3$ are compared in Supplementary Fig. 14 as follow. The film thickness of the FLBP-$CsPbBr_3$ (50%) is thinner than FLBP-$CsPbBr_3$ (75%). Thus, a smaller on/off ratio of FLBP-$CsPbBr_3$ (50%) based device is obtained in the *I-V* characterization in comparison with the FLBP-$CsPbBr_3$ (75%) based device.

## Supplementary Note 5

The substantial output signals shown in Supplementary Fig. 21 suggests the onset of a switching event. It was discovered that at a higher temperature, the early switching event will be triggered. Supplementary Fig. 22 plot temperature dependent characteristics of devices by applying fixed 0.2 V voltage pulses (50 μs in width, 50 μs in interval). The current increases with temperature in the pre-switching state, suggesting a thermally activated electron hopping mechanism[1]. After occurrence of switching event, the current value decreases as the temperature increases, suggesting that the conduction mechanism follows a metallic behavior. This result implies that there would be residual Ag clusters existed between Ag electrode and ITO electrode to reduce the effective gap distance, which can effectively engineer the conduction following a thermal-assistant hopping mechanism.

## Supplementary Note 6

Supplementary Fig. 23 shows the output current as a function of light wavelength and the incident angles. By using the least-squares method with the MATLAB software, the desired output current $f(x,y)$ for the TSM device is determined by

$$f(x,y)=a_1+a_2x+a_3y+a_4x^2+a_5xy+a_6y^2+a_7x^3+a_8x^2y+a_9xy^2+a_{10}x^4+a_{11}x^3y+a_{12}x^2y^2+a_{13}x^5+a_{14}x^4y+a_{15}x^3y^2$$

where $x$ is angle, $y$ is wavelength, $a_1$=0.2444 (0.1635, 0.3254), $a_2$=0.07342 (-0.1191, 0.266), $a_3$=-0.3349 (-0.392, -0.2777), $a_4$=-0.2742 (-0.4382, -0.1102), $a_5$=0.01547 (-0.06, 0.09095), $a_6$=0.1546 (0.09387, 0.2154), $a_7$=-0.1272 (-0.4194, 0.165), $a_8$=0.2437 (0.103, 0.3844), $a_9$=-0.01567 (-0.1228, 0.0915), $a_{10}$=0.07682 (0.01436, 0.1393), $a_{11}$=-0.003562 (-0.04078, 0.03365), $a_{12}$=-0.06462 (-0.1099, -0.01931), $a_{13}$=0.04099 (-0.05244, 0.1344), $a_{14}$=-0.04444 (-0.09662, 0.007733), $a_{15}$=0.002848 (-0.04929, 0.05498).

The light wavelength and incident light can be well decoupled according to the above function.

## Supplementary References

1. Yoon, J. et al. An artificial nociceptor based on a diffusive memristor. *Nat. Commun.* **9**, 417 (2018).
