# Supplementary material for: Memristor-based biomimetic compound eye for real-time collision detection
Source: Nat Commun. 2021 Oct 13;12:5979. doi: 10.1038/s41467-021-26314-8 (PMC8514515; doi:10.1038/s41467-021-26314-8)
Supplement: Supplementary file 3 — Description of Additional Supplementary Files [file 41467_2021_26314_MOESM3_ESM.pdf]

Description of Additional Supplementary Files

File Name: Supplementary Movie 1
Description: The car goes straight under dark condition.

File Name: Supplementary Movie 2
Description: The car turns with an angle with the neuron fire frequency over 2.5 Hz when the light power is 1 mw.

File Name: Supplementary Movie 3
Description: The car goes straight with the neuron fire frequency less than 2.5 Hz when the light power 2.5mw.
